# Verbal fluency patterns associated with the amnestic conversion from mild cognitive impairment to dementia

**DOI:** 10.1038/s41598-024-52562-x

**Published:** 2024-01-23

**Authors:** Simona Cintoli, Laura Favilli, Riccardo Morganti, Gabriele Siciliano, Roberto Ceravolo, Gloria Tognoni

**Affiliations:** 1 Department of Medical Specialties - Neurology Unit, AOUP, 56126 Pisa, Italy; 2 Department of NEUROFARBA- Section of Psychology, University of Florence, 50135 Florence, Italy; 3 Department of Clinical and Experimental Medicine, Section of Statistics, University of Pisa, 56126 Pisa, Italy

**Keywords:** Psychology, Neurology

## Abstract

Patients with amnestic mild cognitive impairment (aMCI) are at a higher risk of converting to Alzheimer's disease. The aim of this study was to examine the potential use of Verbal Fluency (VF) measures as markers for predicting the conversion to dementia. At baseline, 61 aMCI, aged 65 to 80 years, underwent a comprehensive neuropsychological assessment, including phonemic (PVF) and semantic verbal fluency (SVF) tasks. After 18 months, 14 individuals with aMCI had progressed to a diagnosis of dementia. The findings revealed that aMCI-converter group had lower Mini Mental State Examination and Rey Auditory Verbal Learning Task scores than aMCI-no converter and produced fewer clusters in both VF tasks and a lower number of switches in PVF at baseline (*p* < 0.05). According to receiver operating characteristic curve analysis, the number of clusters in PVF had the highest predictive value (AUC = 0.80) with a threshold of 5.510 for identifying aMCI-converter at baseline. Additionally, participants with higher levels of education exhibited more clusters and switches in VF tasks (*p* < 0.05). These results suggest that qualitative measures of VF could serve as neuropsychological markers for predicting cognitive decline in individuals with aMCI. Furthermore, the study highlights the potential influence of the education level on cognitive performance in neuropsychological tasks.

## Introduction

Mild cognitive impairment (MCI) often represents a transitional period between normal cognitive aging and early dementia^1^. Individuals meeting the criteria for MCI have a slight but noticeable and measurable decline in one or multiple cognitive domains, but these deficits are not severe enough to significantly interfere with daily life activities and warrant a diagnosis of dementia^2–5^. A person with MCI has an increased risk of developing Alzheimer's disease (AD) or another form of dementia^6,7^: the rate of yearly progression to dementia in MCI subjects, particularly in the amnestic subtype of MCI (aMCI), is higher than in non-MCI older adults^4,8^. Therefore, emphasis has often been placed on memory deficits. However, recent studies observe that VF (Verbal Fluency) may be an accurate and efficient tool to discriminate between normal aging, a-MCI, and AD^9^. Furthermore, some findings show that the VF task is predictive of incident cognitive impairment and the transition to dementia^10,11^. The predictive accuracy of fluency tests can vary depending on the length of the follow-up. This indicates that its predictive value changes with the disease stage, unlike other tests, such as verbal memory, for which the predictive accuracy remains high and stable over^12^, consistent with other findings that demonstrate the total immediate recall score on the Selective Reminding Test and the Digit Symbol Test coding to be a stronger predictor of conversion over time within a comprehensive neuropsychological battery^13^. Therefore, the analysis of verbal fluency skills in preclinical forms of dementia, such as AD, is crucial both diagnostically and predictively.

VF is defined as the ability to produce words under specific constraints within a fixed time interval^14^. Usually, in clinical and experimental assessments, neuropsychologists use phonemic fluency, which requires the ability to rapidly name words that begin with a specific letter, and semantic fluency, which requires the capacity to produce words that belong to a specific category. The performance on the VF test is measured by counting the number of correct words said by the patient in one minute.

Both fluency tasks require strategic search and information retrieval from semantic memory: semantic fluency requires a constrained search of words from a superordinate category, whereas phonemic fluency may be accomplished with a less constrained search from a larger set of lexical exemplars^15^.

There is evidence in the literature that the verbal fluency task is multifactorial, and the total number of words is not enough to capture all aspects of performance, identify the underlying deficit, and facilitate diagnostic procedures^16,17^.

Qualitative aspects of lexical retrieval can offer additional insights that guide performance on VF tasks^18^. According to Troyer and colleagues, clustering and switching are the two main strategies of VF used to search, organize, and retrieve^16^. In particular, clustering is an executive-linguistic subprocess related to semantic verbal memory, word storage, and refers to the generation of words within a given subcategory^19^; on the other hand, switching refers to the ability to change from one subcategory to another and is related to cognitive flexibility and word process search^19,20^. Both components involve complex cognitive processes such as cognitive flexibility, and they are equally relevant for adequate performance on VF tasks^21^.

Some studies have investigated the effect of age, gender, and educational level on these qualitative components. The age effect is not uniform, considering that different categories have different decreasing tendencies^22^, with young adults often producing more words and switching more frequently than older subjects^23^.

Regarding the effect of gender, according to some studies, males seem to have higher scores on the semantic VF test^22^ and produce larger clusters, whereas females generate a greater number of switches^23^. However, not all studies have replicated these results. In fact, other studies found no effects of gender on both verbal fluency tasks^24,25^. There was also a significant difference between groups with different levels of education: people with higher education had a larger mean cluster size on the PVF task and switched more often in category fluency^19,22,24^. Cognitive reserve is associated in some research with better performance on the verbal fluency test^26,27^. Overall, the prognostic role of this analysis is not yet clear.

Moreover, some studies have investigated various aspects of the VF task to distinguish patients with different levels of cognitive impairment, such as aMCI or AD patients, from healthy older adults with no cognitive decline^28–31^. Some studies showed that switching and clustering capacity in letter and semantic fluency could be important to study in MCI subjects also to predict the conversion to dementia. The word lists generated by the fluency test are a rich source of information to foresee outcomes in MCI^32^. In particular, the shifting abilities could be involved in the early decline in semantic fluency performance in the prodromic phase of AD^33^ and clustering could be used to estimate dementia risk in cognitively normal individuals, especially when looking at the size of semantic clusters^34^.

Considering these observations, the qualitative analysis of verbal fluency tests could allow the examiner to detect some executive dysfunctions in prodromal disease conditions, but further research is needed to assess the applicability of such analysis in different categories and its predictive value. The aim of this paper is to examine the cluster size, the number of clusters, and switches, in addition to the number of correct words, in two VF tasks, phonemic and semantic fluency tests, with six subcategories, to determine which of these measures can sensitively distinguish the aMCI-converter group from the aMCI-no converter group to dementia.

## Methods

### Participants

Subjects were recruited from the general population at the Center for Cognitive Disorders and Dementia in Pisa. The eligible population for the study included older individuals aged between 65 and 80 with at least five years of education and with Mild Cognitive Impairment (MCI) in accordance with the recommendations of the National Institute on Aging and the Alzheimer's Association on diagnostic guidelines for preclinical stages^2,3^; specifically, only amnestic single-domain MCI individuals with normal performance in other cognitive domains were included in the study. Exclusion criteria comprised neurological pathologies, such as dementia, epilepsy, advanced neoplasia, recent cranial trauma, drug addiction, clinical evidence of depression, or other psychiatric disorders. The subjects provided written informed consent for participation, along with information on privacy and the treatment of sensitive data. The study protocol received approval from the Regional Ethical Committee for Clinical Experimentation (Comitato Etico di Area Vasta Nord Ovest—CEAVNO) for the publication of aggregated and anonymously reviewed data collected from medical records without an experimental procedure. The study adhered to the ethical guidelines of the 1975 Declaration of Helsinki.

### Cognitive assessment

We administered a comprehensive battery of neuropsychological tasks to assess performance in various cognitive domains. In particular, cognitive evaluation was conducted using the following tests: Mini Mental State Examination (MMSE)^35^, widely used for screening cognitive impairment and estimating his severity as well as the need for further evaluation; short term memory was evaluated with Babcock Short Story (BSS)^36^ immediate recall (IR, immediate repetition after reading by the experimenter), Rey Auditory Verbal Learning Task (RAVLT)^37^ immediate recall (IR, immediate repetition after reading by the experimenter), the Rey-Osterrieth Complex Figure Test (ROCF)^36^ immediate recall (IR, 1 min after copy), digit span forward^38^ and Corsi block-tapping test forward^38^; retrospective memory was assessed using Babcock Short Story (BSS)^36^ delayed recall (DR, 20 min after the reading in the immediate recall), Rey Auditory Verbal Learning Task (RAVLT)^37^ delayed recall (DR, 15 min after the last reading in the immediate recall) and the Rey-Osterrieth Complex Figure Test (ROCF)^36^, delayed recall (DR, 20 min after the copy); Phonemic Verbal Fluency (PVF)^37^, Semantic Verbal Fluency (SVF)^39^ and Attentional Matrices (AM)^40^ were used to assess executive functions and attention; visuo-spatial abilities and constructional praxis was measured with the copy of Rey-Osterrieth Complex Figure Test (ROCF)^36^. The same neuropsychological assessment was administered after 18 months to determine the clinical conversion. All subjects were native Italian speakers, not bilingual, and were tested in their primary language, which is Italian.

### Verbal fluency qualitative analysis

Verbal fluency is a cognitive function that facilitates information retrieval from memory. Successful retrieval requires executive control over cognitive processes such as selective attention, mental set shifting, internal response generation, and self-monitoring. Tests of verbal fluency evaluate an individual's ability to retrieve as many words as possible from a given category, either semantic or phonemic, within a given time frame (typically 60 s). In these tasks, participants were asked to name as many words as possible that begin with the letters “f”, “a’’, and “s” for PVF^37^, and words that belong to the categories “car brands”, “fruits”, and “animals” for SVF^39^. These are the three standard categories used to measure performance in the semantic fluency task for the Italian population. Participants were instructed to avoid using proper nouns (e.g., Roma, Roberto) and words that begin with the same sound but have different suffixes (e.g., love, lover, loving); errors of this nature were excluded from the total count of correct words.

In this paper, we analyzed several qualitative aspects of verbal production, including: number of switches, this represents the number of transitions between clustered or non-clustered words, including single words; number of clusters, this measures the number of multiword strings, with each cluster containing at least two successive words; mean cluster size, this is calculated as the total cluster size divided by the number of clusters. For each task, we also calculated the total number of words generated with errors and repetitions.

The qualitative aspects of verbal production for letter word fluency were scored using the exact system described by Ledoux and colleagues^18^. We utilized their letter and category scoring guidelines and customized the semantic categories “car brands”, “fruits” and “animals” to align with Italian culture and common usage. Refer to Appendix A for the adjusted subcategories for “car brands”, “fruits” and “animals”, which include frequently generated examples for each category (Supplementary file). Please note that these listings are not exhaustive; for example, we did not consider possible categorizations such as “electric cars” versus “fuel cars”, as brands that exclusively produce electric cars like “Tesla” were not mentioned. The clustering analysis for categories was constructed based on the country of origin according to the analyzed verbal production.

Verbal productions were scored by the same rater, and an additional trained rater, using the guidelines presented in Table 1 by Ledoux and colleagues^18^.

**Table 1:** Baseline characteristics of aMCI subjects; data are expressed as frequency (%) or mean (sd).

	aMCI-no converter (n = 53)	aMCI-converter (n = 14)	*p*-value
Age at the baseline	73.5 (3.9)	74.1 (2.8)	0.627
Gender, women	24 (45.3)	7 (50)	0.753
Education, years	10.7 (4.4)	9.4 (3.3)	0.236

*p* < 0.05.

### Statistical analysis

Continuous data were described by mean and standard deviation. To compare the group variable (aMCI-no converter, aMCI-converter) with continuous factors, we applied a two tailed t-test for independent samples. Additionally, ROC analysis was performed to identify the best cut-off point for the factors that resulted significant. Sensitivity and specificity were also calculated. Furthermore, we examined the influence of age, gender and education on the significant clinical outcomes (SVF number cluster, PVF number cluster and switch) through a multiple linear regression analysis. A significance level of 0.05 was used, and all analyses were conducted with SPSS v.28 technology.

## Results

In this study, we included 61 individuals with amnesic MCI unidomain and analyzed demographic data (Table 1), neuropsychological tests results (Table 2), and qualitative data from VF (Table 3).

**Table 2:** Cognitive tests at baseline of aMCI subjects; data are expressed as mean (sd)

	**aMCI-no converter (n = 53)**	**aMCI-converter (n = 14)**	**p-value**
MMSE, score	26.4 (2.1)	24.0 (2.5)	< 0.001*
RAVLT—IR, score	32.3 (7.0)	28.1 (6.1)	0.048*
RAVLT—DR, score	4.5 (2.3)	3.0 (2.0)	0.038*
BSS—IR, score	4.3 (1.9)	3.4 (1.4)	0.072
BSS—DR, score	3.1 (2.4)	2.6 (2.5)	0.469
Digit Span, score	5.5 (0.8)	5.1 (0.9)	0.132
Corsi block-tapping, score	4.7 (0.7)	4.5 (0.5)	0.433
ROCF copy, score	32.4 (3.5)	30.0 (5.5)	0.145
ROCF IR, score	11.8 (5.1)	10.0 (4.0)	0.212
ROCF DR, score	11.1 (5.2)	8.5 (5.5)	0.111
Attentional matrices, score	50.1 (6.6)	47.9 (8.9)	0.303
PVF, score	35.7 (10.8)	29.5 (9.2)	0.054
SVF, score	37.4 (9.4)	31.9 (8.6)	0.052

MMSE, mini mental state examination; RAVLT, Rey auditory verbal learning task; BSS, Babcock short story; ROCF, Rey-Osterrieth complex figure test; PVF, phonemic verbal fluency; SVF, semantic verbal fluency; IR, immediate recall; DR, delayed recall. *p < 0.05.

**Table 3:** Verbal Fluency Qualitative Analysis at baseline of aMCI subjects; data are expressed as mean (SD).

	aMCI-no converter (n = 53)	aMCI-converter (n = 14)	*p*-value
PVF, number cluster	7.1 (2.8)	4.3 (2.0)	0.001*
PVF, cluster size	6.9 (1.6)	6.3 (3.6)	0.554
PVF, number switch	21.2 (7.1)	16.4 (4.9)	0.022*
SVF, number cluster	8.2 (2.5)	6.4 (2.0)	0.009*
SVF, cluster size	7.1 (1.6)	6.9 (1.7)	0.601
SVF, number switch	16.2 (5.3)	13.4 (4.0)	0.065

PVF, phonemic verbal fluency; SVF, semantic verbal fluency. *p < 0.05.

After 18 months from baseline, the subjects underwent a second neuropsychological assessment to evaluate changes in cognitive status. Specifically, 14 individuals with aMCI progressed to a diagnosis of dementia, termed aMCI-converter (MCI-C), while 53 aMCI subjects maintained a stable diagnosis, referred to as aMCI-no converter (MCI-NC).

In detail, of the 14 subjects, who progressed to a diagnosis of dementia, approximately 86% (n = 12) received a diagnosis of Alzheimer's disease based on recommendations from the National Institute on Aging-Alzheimer's Association workgroups^41^, one subject received a diagnosis of Behavioral Variant of Frontotemporal Dementia (bvFTD)^42^, and one of vascular dementia (VaD)^43^.

MCI-C and MCI-NC were similar in terms of age, education, and gender distribution (*p* > 0.05 for all p-values; Table 1).

Both groups displayed a similar neuropsychological profile, except for the MMSE test and the Rey Auditory Verbal Learning Task, in which MCI-NC had a better performance than MCI-C (*p* < 0.05 for both p-values; Table 2). Additionally, MCI-NC demonstrated a trend toward generating more words in both verbal fluency tests (*p* = 0.054 for PVF; *p* = 0.052 for SVF, Table 2).

Furthermore, the two groups showed substantial differences in the qualitative analysis of VF tests. In both fluency tests, PVF and SVF, we found a statistical difference in the number of clusters; in particular, MCI-NC generated more clusters during the tasks (*p* < 0.05 for all p-values; Table 3). In PVF, we observed a statistical difference in the number of switches, and in SVF, MCI-NC showed a trend toward producing more switches (*p* = 0.022 and *p* = 0.065, respectively; Table 3).

ROC analysis was conducted for each significant clustering and switching factor to determine the optimal cut-off value and provide information about the most sensitive and specific neuropsychological tools for predicting the development of dementia. In the PVF test, the number cluster score had the best area under the curve (AUC value = 0.80), also in comparison to other tests such as MMSE, RAVLT-IR and RAVLT-DR (AUC value = 0.788; 0.650; 0.668, respectively). The significant threshold for the subscores of the "PVF number cluster" in identifying MCI-C at baseline was 5.510. The "SVF number cluster" provided high sensitivity of 86%, and the "PVF number cluster" had the highest specificity of 74%. Sensitivity and specificity values are also presented in Table 4.

**Table 4:** ROC analysis was performed for each significant variable to determine the optimal cut-off value.We also calculated AUC, sensitivity and specificity to determine which variables were accurate to predict the clinical diagnosis.

Variable	AUC	95% CI	The best cut-off	Sensitivity (%)	Specificity (%)
PVF, number cluster	0.800	0.677–0.923	5.510	79	74
PVF, number switch	0.712	0.570–0.853	18.500	72	71
SVF, number cluster	0.714	0.579–0.850	7.510	86	62

AUC, Area under ROC curve; CI, confidence interval; PVF, phonemic verbal fluency; SVF, semantic verbal fluency.

We conducted a multiple linear regression analysis to examine factors influencing the significant dependent variables: SVF number cluster, PVF number cluster and switch. In particular, years of education significantly influenced all three dependent variables under examination (*p* < 0.05 for all *p*-values; Table 5).

**Table 5:** Multiple linear regression of the factors influencing several dependent variables.

Variables	RC	95%CI—lower	95%CI—upper	*p*-value
PVF, number cluster				
Age	0.022	− 0.134	0.179	0.775
Gender	0.067	− 1.218	1.352	0.918
Education, years	0.203	0.048	0.358	0.011*
Group (MCI-converter/-no converter)	− 2.541	− 4.084	− 0.998	0.002*
PVF, number switch				
Age	0.170	− 0.161	0.501	0.309
Gender	2.313	− 0.411	5.037	0.095
Education, years	0.990	0.662	1.317	< 0.001*
Group (MCI-converter/-no converter)	− 3.658	− 6.929	− 0.387	0.029*
SVF, number cluster				
Age	− 0.066	− 0.207	0.076	0.357
Gender	− 0.321	− 1.485	0.844	0.584
Education, years	0.198	0.058	0.338	0.006*
Group (MCI-converter/-no converter)	− 1.556	− 2.955	− 0.158	0.030*

PVF, phonemic verbal fluency; SVF, semantic verbal fluency. *p-value < 0.05.

## Discussion

Tests of phonemic and semantic verbal fluency are commonly used in the assessments of individuals with memory complaints and in the clinical diagnosis of Alzheimer's disease (AD) and other types of dementia. Qualitative aspects of lexical retrieval can provide additional insights into the cognitive processes that underlie performance on verbal fluency tasks^18,44^. The mere counting of words generated is not a sufficient distinction between dementia and other cognitive impairments^16^.

According to Troyer and colleagues^29^, clustering and switching are the two main strategies used in verbal fluency tests to search for, organize, and retry words. The objective of our current study was to determine if assessing switching and clustering behavior in verbal fluency tests could yield measurements associated with the risk of developing dementia. Specifically, we aimed to identify potential neuropsychological markers for conversion from amnestic MCI status, where fluency abilities and performance are not clinically impaired, to dementia over an 18-month follow-up period.

At baseline, the Mini Mental State Examination Test (MMSE) and, to a lesser extent, the Rey Auditory Verbal Learning Test (RAVLT), were the only tests that appeared to predict a change in diagnosis. Specifically, the aMCI-converter group (MCI-C) had lower scores than the aMCI-no converter group (MCI-NC) in the MMSE and the RAVLT at both the immediate and delayed recall scores. These findings are in line with existing literature, as the MMSE is a useful tool for screening cognitive decline, and the RAVLT is a powerful test for detecting changes in episodic memory, which are characteristic of aMCI^45^.

The investigation of VF in preclinical forms of dementia, such as AD, is crucial for both diagnostic and predictive purposes. Changes in VF have been observed in individuals years before the clinical diagnosis of AD^46^, and patients with AD typically perform poorly on fluency tests compared to older adults without cognitive decline^47,48^. Moreover, a progressive decline in the number of words generated during fluency tasks has been observed in older populations in both tasks^49,50^ particularly during the transition from middle-age to early elderly^51^.

Verbal fluency's global score, which measures speeded access to lexical and semantic information, has limitations in providing insight into crucial aspects of a subject's performance and underlying cognitive mechanisms, such as cognitive flexibility, search strategy, executive function, working memory, processing speed, and verbal ability. Consequently, some studies have focused on analyzing performance based on clustering and switching. For example, one study found that the number of switches more sensitively distinguished MCI from a control group than the number of correct words^20^. Therefore, qualitative aspects of fluency performances like clustering and switching have been explored^16^.

The results showed that at baseline, aMCI subjects who later converted to dementia produced a lower number of clusters in both VF tasks compared to those who did not convert to dementia. These findings are consistent with previous research that found fewer clusters in MCI subjects compared to a control group^28,30^. MCI-C subjects produced fewer number of clusters, likely because they had deficits in strategic research or the ability to initiate a search for new strategies or subcategories. VF tasks activate common cognitive processes at various levels that are part of a distributed system involved in verbal recall^16,52^. Both phonemic and semantic tasks are associated with frontal structures, but the latter also depends on temporal systems^53,54^.

We also observed a difference in the number of switches in the PVF task and a trend in SVF, possibly due to the smaller number of MCI subjects who converted to dementia.

This is in line with previous studies. For example, Weakley and colleagues^31^ found that multidomain aMCI subjects scored worse in total words and switching production compared with healthy controls on both fluency tasks. Raoux and colleagues^33^ also discovered that there is a significantly lower switching index in future AD subjects, even five years before the dementia diagnosis. Since switching is a measure of cognitive flexibility and involves the ability to access novel information in the semantic memory system^29,55^, difficulties with these processes could contribute to the progression to dementia.

Additionally, we did not find any differences in mean cluster sizes both in PVF and SVF tasks. Some research in fact has shown that AD subjects form smaller clusters than control groups^28,29^, although the mean cluster size may not effectively discriminate future AD subjects^15,33^.

The worse performance in VF at baseline among the MCI group that progressed to mild dementia in the follow-up suggests that phonemic and semantic processes (search and retrieval of information from memory) might be affected in the early stages. These changes could characterize the transition to full-blown disease^56^. In particular, the tendency of subjects who later developed dementia to form smaller clusters could support the hypothesis of disorganization of lexical representations^57^.

Educational level played a significant role in performance, underscoring the importance of considering sociocultural aspects in neuropsychological assessments^21^. Multiple linear regression of demographic variables highlighted that the years of education influenced the qualitative aspects of verbal production, particularly the number of clusters and switches in both VF tasks. This is significant because education is one of the most important predictors of dementia. Higher education is associated with a reduced risk of developing dementia and provides a cognitive reserve against the effects of aging and disease on brain function^58^. Additionally, subjects with higher education appeared to be more flexible in their search for new clusters, a strategy associated with educational stimulation, cognitive flexibility, and cognitive reserve (CR) capacity^21^. This study did not provide evidence of age and gender differences in the strategies used during VF tests.

This manuscript underlines the diagnostic and predictive importance of investigation of verbal fluency in MCI. In particular, the results highlight that a detailed analysis of verbal fluency may be helpful to distinguish patients with mild cognitive impairment who are at risk of developing a form of dementia in the near future. To the best of our knowledge, no studies have investigated two verbal fluency tasks, phonemic and semantic fluency tests, with six subcategories, to determine which of these measures significantly distinguishes patients who convert to a clinical diagnosis of dementia after 18 months. The present study had some limitations that need to be addressed in future research. First, the sample size should be increased to enhance the strength of the analysis and this would be our first aim in the future. Additionally, a longer follow-up period, exceeding 18 months, would provide more accurate results. Future studies could benefit from longitudinal designs that include measurements of brain pathology to investigate the relationship between VF and neurodegeneration.

In conclusion, our findings emphasize the value of conducting a detailed and qualitative analysis of neuropsychological tests, with a specific focus on verbal fluency. This approach can offer valuable insights into early cognitive changes and assist in predicting the progression from amnestic Mild Cognitive Impairment to dementia (Supplementary file). Such investigations are crucial for developing interventions that can effectively delay the onset of dementia.

Moreover, analyzing the performance of verbal fluency tasks in the MCI group provides a deeper understanding of not only the cognitive characteristics of MCI but also the cognitive mechanisms involved in the progression to dementia. This suggests that individual fluency tasks can reveal specific cognitive processes.

Overall, our results highlight the importance of including multiple verbal fluency tests in assessment batteries for preclinical dementia populations. Additionally, the establishment of optimal thresholds for assessing the number of clusters in both VF tasks through ROC analysis, combined with the versatility, data richness, low cost, and quick administration of verbal fluency tests, has the potential to serve as a rapid and valuable tool in neuropsychological assessments as a screening instrument for early detection of neurodegenerative diseases.

### Supplementary Information

Supplementary Information.

## Data Availability

We have endeavored to adhere to the Transparency and Openness Promotion (TOP) Guidelines by the community working group in conjunction with the Center for Open Science^59^ in the preparation and reporting of this study. A clear and detailed description of the study design, including the research method, participants, materials, and procedures, has been provided to ensure full transparency. Ethical approval for this study was obtained from local ethics committee. We collected data from standardized and validated neuropsychological instruments on the target population, specifically described in the Methods section. No data exclusions were made during the analysis. All collected data were included in the final dataset and are available for scrutiny. The data analysis was conducted using SPSS v.28 with appropriate analyses for each research question outlined, to provide a comprehensive view of our research results. The interpretations and conclusions drawn from the data have been presented objectively and transparently, acknowledging any limitations or potential biases.

## References

[1] Cintoli S (2019). Effects of combined training on neuropsychiatric symptoms and quality of life in patients with cognitive decline. Aging. Clin. Exp. Res..

[2] Albert MS (2011). The diagnosis of mild cognitive impairment due to Alzheimer's disease: Recommendations from the National Institute on Aging-Alzheimer's Association workgroups on diagnostic guidelines for Alzheimer's disease. Alzheimers Dement..

[3] Croisile B, Auriacombe S, Etcharry-Bouyx F, Vercelletto M (2012). The new 2011 recommendations of the National Institute on Aging and the Alzheimer's Association on diagnostic guidelines for Alzheimer's disease: Preclinal stages, mild cognitive impairment, and dementia. Rev. Neurol..

[4] Petersen RC (1999). Mild cognitive impairment: Clinical characterization and outcome. Arch. Neurol..

[5] Petersen RC (2018). Practice guideline update summary: Mild cognitive impairment: Report of the Guideline Development, Dissemination, and Implementation Subcommittee of the American Academy of Neurology. Neurology.

[6] Sperling R, Johnson K (2013). Biomarkers of Alzheimer disease: Current and future applications to diagnostic criteria. Continuum (Minneap. Minn.).

[7] Ward DD, Wallace LM, Rockwood K (2021). Frailty and risk of dementia in mild cognitive impairment subtypes. Ann. Neurol..

[8] Palmer K, Bäckman L, Winblad B, Fratiglioni L (2008). Mild cognitive impairment in the general population: Occurrence and progression to Alzheimer disease. Am. J. Geriatr..

[9] McDonnell M (2020). Verbal fluency as a screening tool for mild cognitive impairment. Int. Psychogeriatr..

[10] Gallucci M (2018). Neuropsychological tools to predict conversion from amnestic mild cognitive impairment to dementia. The TREDEM Registry. Neuropsychol. Dev. Cogn. B. Aging Neuropsychol. Cogn..

[11] Sutin AR, Stephan Y, Terracciano A (2019). Verbal fluency and risk of dementia. Int. J. Geriatr. Psychiatry.

[12] Belleville S (2017). Neuropsychological measures that predict progression from mild cognitive impairment to Alzheimer's type dementia in older adults: A systematic review and meta-analysis. Neuropsychol. Rev..

[13] Tabert MH (2006). Neuropsychological prediction of conversion to Alzheimer disease in patients with mild cognitive impairment. Arch. Gen. Psychiatry..

[14] Lezak MD, Howieson DB, Loring DW, Fischer JS (2004). Neuropsychological Assessment.

[15] Murphy KJ, Rich JB, Troyer AK (2006). Verbal fluency patterns in amnestic mild cognitive impairment are characteristic of Alzheimer's type dementia. Int. Neuropsychol. Soc..

[16] Troyer AK, Moscovitch M, Winocur G (1997). Clustering and switching as two components of verbal fluency: Evidence from younger and older healthy adults. Neuropsychology.

[17] Wong MN, Murdoch B, Whelan BM (2010). Language disorders subsequent to mild traumatic brain injury (MTBI): Evidence from four cases. Aphasiology.

[18] Ledoux K (2014). Capturing additional information about the organization of entries in the lexicon from verbal fluency productions. J. Clin. Exp. Neuropsychol..

[19] Troyer AK (2000). Normative data for clustering and switching on verbal fluency tasks. Clin. Exp. Neuropsychol..

[20] Oh SJ, Sung JE, Choi SJ, Jeong JH (2019). Clustering and switching patterns in semantic fluency and their relationship to working memory in mild cognitive impairment. Dement. Neurocogn. Disord..

[21] Pereira T (2018). Neuropsychological predictors of conversion from mild cognitive impairment to Alzheimer’s disease: A feature selection ensemble combining stability and predictability. BMC Med. Inform. Decis. Mak..

[22] Van der Elst W, Van Boxtel MP, Van Breukelen GJ, Jolles J (2006). Normative data for the Animal, Profession and Letter M Naming verbal fluency tests for Dutch speaking participants and the effects of age, education, and sex. J. Int. Neuropsychol. Soc..

[23] Lanting S, Haugrud N, Crossley M (2009). The effect of age and sex on clustering and switching during speeded verbal fluency tasks. J. Int. Neuropsychol. Soc..

[24] Brucki SM, Rocha MS (2004). Category fluency test: Effects of age, gender and education on total scores, clustering and switching in Brazilian Portuguese-speaking subjects. Braz. J. Med Biol. Res..

[25] Mathuranath PS (2003). Effects of age, education and gender on verbal fluency. J. Clin. Exp. Neuropsychol..

[26] Lavrencic LM, Churches OF, Keage HA (2018). Cognitive reserve is not associated with improved performance in all cognitive domains. Appl. Neuropsychol. Adult..

[27] Roldán-Tapia L, García J, Cánovas R, León I (2012). Cognitive reserve, age, and their relation to attentional and executive functions. Appl. Neuropsychol. Adult..

[28] Fagundo AB (2008). Clustering and switching in semantic fluency: Predictors of the development of Alzheimer's disease. Int. J. Geriatr. Psychiatry..

[29] Troyer AK, Moscovitch M, Winocur G, Leach L, Freedman M (1998). Clustering and switching on verbal fluency tests in Alzheimer's and Parkinson's disease. J. Int. Neuropsychol. Soc..

[30] Price SE (2012). Semantic verbal fluency strategies in amnestic mild cognitive impairment. Neuropsychology.

[31] Weakley A, Schmitter-Edgecomb M, Anderson J (2013). Analysis of verbal fluency ability in amnestic and non-amnestic mild cognitive impairment. Arch. Clin. Neuropsychol..

[32] Clark LJ (2009). Longitudinal verbal fluency in normal aging, preclinical, and prevalent Alzheimer's disease. Am. J. Alzheimers Dis. Other Demen..

[33] Raoux N (2008). Clustering and switching processes in semantic verbal fluency in the course of Alzheimer's disease subjects: Results from the PAQUID longitudinal study. Cortex.

[34] Pakhomov SV, Hemmy LS (2014). A computational linguistic measure of clustering behavior on semantic verbal fluency task predicts risk of future dementia in the nun study. Cortex.

[35] Magni E, Binetti G, Bianchetti A, Rozzini R, Trabucchi M (1996). Mini-Mental State Examination: A normative study in Italian elderly population. Eur. J. Neurol..

[36] Carlesimo GA (2002). Standardizzazione di due test di memoria per uso clinico: Breve Racconto e Figura di Rey. Riv. Neurol..

[37] Carlesimo GA (1996). The mental deterioration battery: Normative data, diagnostic reliability and qualitative analyses of cognitive impairment. Eur. Neurol..

[38] Orsini A (1987). Verbal and spatial immediate memory span: Normative data from 1355 adults and 1112 children. Ital. J. Neurol. Sci..

[39] Novelli G, Papagno C, Capitani E, Laiacona M (1986). Tre test clinici di memoria verbale a lungo termine: Taratura su soggetti normali. Arch. Psicol. Neurol. Psichiatr..

[40] Spinnler H, Tognoni G (1987). Italian standardization and classification of Neuropsychological tests. The Italian Group on the Neuropsychological Study of Aging. Ital. J. Neurol. Sci. Suppl..

[41] McKhann GM (2011). The diagnosis of dementia due to Alzheimer's disease: Recommendations from the National Institute on Aging-Alzheimer's Association workgroups on diagnostic guidelines for Alzheimer's disease. Alzheimers Dement..

[42] Rascovsky K (2007). Diagnostic criteria for the behavioral variant of frontotemporal dementia (bvFTD): Current limitations and future directions. Rev. Alzheimer Dis. Assoc. Disord..

[43] Sachdev RK (2014). Internationlal society for vascular behavioral and cognitive. Disorders Diagnostic criteria for vascular cognitive disorders: A VASCOG statement. Alzheimer Dis. Assoc. Disord..

[44] Zhao Q, Guo Q, Hong Z (2013). Clustering and switching during a semantic verbal fluency test contribute to differential diagnosis of cognitive impairment. Neurosci. Bull..

[45] Balthazar ML, Yasuda CL, Cendes F, Damasceno BP (2010). Learning, retrieval, and recognition are compromised in aMCI and mild AD: are distinct episodic memory processes mediated by the same anatomical structures?. J. Int. Neuropsychol. Soc..

[46] Bäckman L, Jones S, Berger AK, Laukka EJ, Small BJ (2005). Cognitive impairment in preclinical Alzheimer's disease: A meta-analysis. Neuropsychology.

[47] Gomez RG, White DA (2006). Using verbal fluency to detect very mild dementia of the Alzheimer type. Arch. Clin. Neuropsychol..

[48] Haugrud N, Crossley M, Vrbancic M (2011). Clustering and switching strategies during verbal fluency performance differentiate Alzheimer's disease and healthy aging. J. Int. Neuropsychol. Soc..

[49] Hills TT, Mata R, Wilke A, Samanez-Larkin GR (2013). Mechanisms of age-related decline in memory search across the adult life span. Dev. Psychol..

[50] Loonstra AS, Tarlow AR, Sellers AH (2001). COWAT metanorms across age, education, and gender. Appl. Neuropsychol..

[51] Gonzalez-Burgos L, Hernández-Cabrera JA, Westman E, Barroso J, Ferreira D (2019). Cognitive compensatory mechanisms in normal aging: A study on verbal fluency and the contribution of other cognitive functions. Aging.

[52] Kavé G, Heled E, Vakil E, Agranov E (2011). Which verbal fluency measure is most useful in demonstrating executive deficits after traumatic brain injury?. J. Clin. Exp. Neuropsychol..

[53] Birn RM (2010). Neural systems supporting lexical search guided by letter and semantic category cues: A self-paced overt response fMRI study of verbal fluency. Neuroimage.

[54] Henry JD, Crawford JR, Phillips LH (2004). Verbal fluency performance in dementia of the Alzheimer’s type: A meta-analysis. Neuropsychologia.

[55] Nutter-Upham KE (2008). Verbal fluency performance in amnestic MCI and older adults with cognitive complaints. Arch. Clin. Neuropsychol..

[56] Venneri A, Mitolo M, De Marco M (2016). Paradigm shift: Semantic memory decline as a biomarker of preclinical Alzheimer's disease. Biomark. Med..

[57] Nikolai T (2018). Semantic verbal fluency impairment is detectable in patients with subjective cognitive decline. Appl. Neuropsychol. Adult.

[58] Fratiglioni L, Wang HX (2007). Brain reserve hypothesis in dementia. J. Alzheimers Dis..

[59] Nosek BA (2015). SCIENTIFIC STANDARDS. Promoting an open research culture. Science.

