# Supplementary material for: Verbal fluency patterns associated with the amnestic conversion from mild cognitive impairment to dementia
Source: Sci Rep. 2024 Jan 23;14:2029. doi: 10.1038/s41598-024-52562-x (PMC10806051; doi:10.1038/s41598-024-52562-x)
Supplement: Supplementary file 1 — Supplementary Information. [file 41598_2024_52562_MOESM1_ESM.docx]

## **SUPPLEMENTAL MATERIALS**

**Appendix A.** Examples are provided of common subcategories and their members for “car brands”, “fruits” and “animals”.

| **CAR BRANDS** | |  |
| --- | --- | --- |
| **Subcategory** | **Examples** |  |
| Czech Republic | Skoda, MW Motors |  |
| France | Citroen, Bugatti, Peugeot, Renault, Simca |  |
| Germany | Audi, Bmw, Mercedes, Opel, Porsche, Smart, Volkswagen |  |
| Italy | Abarth, Alfa Romeo, Aprilia, Bianchi, Ducati, Ferrari, Fiat, Innocenti, Lamborghini, Lancia, Maserati |  |
| Japan | Daihatsu, Honda, Lexus, Mazda, Mitzubishi, Nissan, Subaru, Suzuki, Toyota |  |
| Romania | Dacia, Daewoo |  |
| South Korea | Hyundai, Kia |  |
| Spain | Seat, Pegaso |  |
| Sweden | Saab, Volvo |  |
| United Kingdom | Bentley, Jaguar, Land Rover, Mini, Morris, Rolls Royce, Talbot |  |
| United States | Cadillac, Chevrolet, Chrysler, Daiw, Dodge, Ford, Jeep, Lincoln, Tesla |  |
| Luxury car | Bentley, Ferrari, Jaguar, Lamborghini, Porsche, Rolls Royce |  |
| Utility car | Citroen, Dacia, Fiat, Kia, Hyundai, Mazda, Nissan, Opel, Peugeot, Renault, Saab, Seat, Skoda, Suzuki, Tata, Toyota, Volkswagen |  |
| **FRUITS** | | |
| **Subcategory** | | **Examples** |
| Citrus fruits | | Cedar, lemon, lime, tangerine, orange, grapefruit |
| Dried fruit | | Almonds, hazelnuts, peanut, walnuts, wot |
| Exotic fruit | | Avocado, coconut, date, guaranà, mango, maracuya, papaya, pineapple |
| Berry | | Blueberry, blackberry, raspberry, strawberry |
| Spring/Summer | | Apricot, cherry, coconut, kiwi, melon, peach, plum, watermelon, strawberry |
| Autumn/Fall | | Apple, grape, tangerine, orange, pear, pumpkin, chestnut |
| Orange fruit | | Grapefruit, orange, tangerine |
| Red fruit | | Cherry, raspberry, strawberry |
| Yellow fruit | | Cedar, lemon, banana |
| **ANIMAL** | | |
| **Subcategory** | | **Examples** |
| African | | antelope, baboon, buffalo, camel, cheetah, chimpanzee, cobra, dromedary, elephant, gazelle, giraffe, gnu, gorilla, hippopotamus, hyena, jackal, lemur, leopard, lion, manatee, meerkat, mongoose, monkey, ostrich, panther, rhinoceros, tiger, wildebeest, warthog, zebra |
| Arctic | | Penguin, polar bear, reindeer, seal |
| Australian | | Dingo, emu, kangaroo, kiwi, koala, platypus, Tasmanian devil, wallaby |
| Bird | | Canary, condor, crane, eagle, finch, hawk, parrot, pelican, penguin, pigeon, robin, toucan, woodpecker |
| Bovine | | Bison, buffalo, bull, cow |
| Camels | | Alpaca, camel, dromedary, guanaco, llama |
| Canine | | Coyote, dog (bulldog, dalmatian, chiwawa, etc.), fox, hyena, jackal, wolf |
| Deer | | Antelope, caribou, eland, elk, gazelle, gnu, impala, moose, reindeer, wildebeest |
| Equine | | Donkey, horse, mule, pony, zebra |
| Farm | | Boar, Chicken, cow, donkey, duck, goat, goose, horse, mule, pig, rabbit, sheep, sow, turkey, rooster, ox |
| Feline | Cat, cheetah, cougar, jaguar, leopard, lion, lynx, ocelot, panther, tiger |  |
| Fish | Bass, gilthead bream, goldfish, guppy, manta, ray, salmon, shark, trout |  |
| In pet store | Bird, canary, cat, dog, ferret, fish, guinea pig, hamster, lizard, parrot, rabbit, rat, snake, turtle |  |
| Insects | Ant, beetle, butterfly, cockroach, flea, fly, praying mantis, mosquito, spider |  |
| Primates | Ape, baboon, chimpanzee, gibbon, gorilla, lemur, marmoset, monkey, orangutan |  |
| Reptiles | Alligator, chameleon, crocodile, frog, gecko, iguana, lizard, salamander, snake, toad, turtle |  |
| Rodents | Beaver, capybara, chinchilla, chipmunk, coney, guinea pig, hamster, hare, marmot, mole, mouse, porcupine, rabbit, raccoon, rat, squirrel |  |
| Used for fur | | Beaver, chinchilla, fox, mink, raccoon, ox |
